# Korean colonoscopy screening pilot study (K-cospi) for screening colorectal cancer: study protocol for the multicenter, community-based clinical trial

**DOI:** 10.1186/s12876-021-01610-1

**Published:** 2021-01-26

**Authors:** Bomi Park, Jae Kwan Jun, Byung Chang Kim, Kui Son Choi, Mina Suh, The Expert Advisory Committee, Young Sun Kim, Tae Il Kim, Eun-Cheol Park, Dae Kyung Sohn, JinHee Sohn, Eui Gon Youk, Woong Ki Chang, Byung Ik Jang, Seung-Yong Jeong, Jae Myung Cha, Jae Yong Han, The Monitoring Committee, Byung Chang Kim, Hyun Soo Kim, Aesun Shin, Jae Kwan Jun, Nam-Kyong Choi, Center for Korean Colonoscopy Screening Pilot Study, Mina Suh, Bun Kim, Seung-Kwon Myung, Bomi Park, Ji Young Lee, Kui Son Choi, Kyung Su Han, The Research Team on the Protocol Development of Pilot study, Seung-Yong Jeong, Jong-Phil Lim, Aesun Shin, Ji Won Park, Dae Kyung Sohn, Byung Chang Kim, Mina Suh, Yeol Kim, Sunho Choi, Kyeongmin Lee, Haejoo Seo

**Affiliations:** 1 National Cancer Control Institute, National Cancer Center, 323 Ilsan-ro, Ilsandong-gu, Goyang, Korea; 2 Graduate School of Cancer Science and Policy, National Cancer Center, Goyang, Korea; 3 Center for Colorectal Cancer, Research Institute and Hospital, National Cancer Center, Goyang, Korea; 4 Center for Cancer Prevention and Early Detection, Research Institute and Hospital, National Cancer Center, Goyang, Korea

**Keywords:** Colonoscopy, Colorectal cancer screening, Protocol, Pilot study, National Cancer Screening Program

## Abstract

**Background:**

The Korean National Cancer Screening Program has been providing annual fecal immunochemical test for colorectal cancer (CRC) to adults aged 50 years and older since 2004. The Korean Colonoscopy Screening Pilot Study was developed to evaluate the effectiveness of colonoscopy screening for CRC incidence and mortality, screening-related complications, and acceptability of colonoscopy as a primary modality for the national CRC screening program.

**Methods:**

This study and its protocols have been approved by the Korean Public Institutional Review Board and the National Cancer Center Institutional Review Board. We obtain written informed consent from all participants. The target population is males and females aged 50–74 years living within the pilot sites. A total of 26,640 participants will be recruited for colonoscopy screening. Subjects who have been diagnosed with CRC, who are currently undergoing treatment for CRC, or who have undergone colonoscopy screening within the past 5 years are not allowed to participate. All participants need to complete baseline questionnaires. This pilot study is currently conducted by 104 endoscopists from 57 national cancer screening institutions (42 primary, 10 secondary, and 5 tertiary institutions) located in Goyang-si, Gimpo-si, and Paju-si. The number of endoscopists, medical institutions, and districts participating in the pilot study will be expanded, if necessary. Participating endoscopists at each medical institution perform colonoscopy and report the colonoscopy results to a centralized electronic case report system. We conduct a telephone survey after 7 days and 4 weeks post-colonoscopy to assess for procedure-related complications and satisfaction of the participants. In case of abnormal findings from colonoscopy screening, we track the results from follow-up diagnostic tests. Data from this pilot study will be linked to the diagnostic workup results, the Korean Cancer Registry, and death certificate data for analysis of the performance, long-term effects, and cost-effectiveness of colonoscopy.

**Discussion:**

The results will provide critical information to determine whether the introduction of colonoscopy as the primary modality of the Korean National Cancer Screening Program would be acceptable and feasible.

*Trial registration* Korean Clinical Research Information Service registry, KCT0004142. Registered on 15 July 2019, http://cris.nih.go.kr/cris/en/search/search_result_st01.jsp?seq=16227

## Background

Colorectal cancer (CRC) was responsible for more than 10% of the global cancer burden in 2018 [1]. It is the third most common cancer in men and the second most common cancer in women worldwide, with more than 730,000 new cases being diagnosed each year [2]. CRC has a high burden of disease in Korea as well as the second most common cancer and the third most common cause of cancer-related deaths in men and women combined [3].

CRC starts as a locally confined polypoid precursor lesion and has a long preclinical stage, with most symptoms presented at an advanced stage. Morbidity and mortality of CRC can be significantly reduced if the disease is diagnosed and treated at an earlier stage [4, 5]. Therefore, screening for CRC before the development of symptoms may be beneficial for improved long-term outcomes. There are several screening modalities for CRC, which include fecal occult blood test (FOBT), sigmoidoscopy, colonoscopy, computed tomography colonography, and stool or blood DNA test. Colonoscopy allows for the detection and removal of early cancer and precancerous adenoma in one-step, and thus, it may have a large effect on decreasing CRC incidence and mortality.

Since 2004 as part of the National Cancer Screening Program (NCSP), the government of the Republic of Korea has been providing annual fecal immunochemical test (FIT) for free to adults aged 50 years and older as the primary modality for detecting CRC. Individuals with a positive FIT result are then referred for either colonoscopy or double-contrast barium enema, which are both provided free-of-charge. However, 68.7% of the patients preferred colonoscopy as the primary method for CRC screening [6]. There has been consistent demand for the introduction of colonoscopy as the primary method for the national CRC screening program. However, colonoscopy is an invasive and expensive procedure. In addition, it entails a risk of complications, requires extensive bowel preparation, and is difficult to learn. Therefore, the benefits and harms of colonoscopy should be carefully evaluated. Currently, we lack population-based clinical trials investigating both the magnitude of effectiveness and the harms of colonoscopy screening for CRC.

For these reasons, the Korean Ministry of Health and Welfare commissioned the Korean Colonoscopy Screening Pilot Study (K-cospi) to the National Cancer Center in January 2019 to evaluate the appropriateness and effectiveness of colonoscopy as the primary method for organized CRC screening at the population level. Accordingly, a Center for K-cospi was established in the National Cancer Center in April 2019 to develop and implement the pilot study protocol, execute the budget, facilitate the participation of eligible participants, provide ongoing support to the participating medical institutions, collect data, and evaluate the outcomes.

The Expert Advisory Committee composed of multidisciplinary experts from various fields of CRC screening, including gastroenterology, surgery, pathology, epidemiology, and preventive medicine, who are representatives from medical colleges, professional societies, or primary medical care was established to provide advice on the design and implementation of the pilot study, monitor the overall process, and ensure quality assurance of the study. In addition, the Monitoring Committee, composed of experts in either cancer screening or colonoscopy, was established to regularly monitor the operating procedures, outcomes, and safety of the pilot study.

### Aim

The primary objective of this study is to evaluate the effectiveness of colonoscopy screening for CRC incidence and mortality in the Republic of Korea. The secondary objectives of this study are to evaluate the complications related to colonoscopy screening and to assess the acceptability of colonoscopy for CRC screening.

## Methods

### Study design and setting

This is a multicenter, community-based clinical trial to evaluate the effectiveness, harms, and acceptability of colonoscopy screening for CRC. The criteria for the selection of pilot sites included

- Sufficient number of the target population;
- Epidemiologic characteristics of CRC, which represent the Korean population;
- Mixture of urban and rural characteristics to minimize the impact of specific regional characteristics on the study results;
- Even distribution of medical institutions capable of colonoscopy screening;
- Enough endoscopists and endoscopy units to be involved in the pilot study;
- Support and cooperation from local governments for the promotion and implementation of the pilot program.

Goyang-si, Gimpo-si, and Paju-si in Korea satisfied the above criteria and were selected as the pilot sites (Paju-si was additionally included in January 2020).

Medical institutions located in the pilot sites and designated as CRC screening units by the National Health Insurance Service were allowed to participate in the pilot study. They were designated as CRC screening units after undergoing a verification process to ensure that they met the application qualification and were equipped with the necessary manpower, facilities, and equipment. The verified institutions will be frequently checked for the management of equipment and facilities, in accordance with the Framework Act on Health Examination.

The essential prerequisites for endoscopists to participate in the pilot study were as follows:

- Employed at the medical institutions participating in the pilot study;
- Certified for endoscopy from either of the Korean Society of Gastrointestinal Endoscopy, the Korean Society of Digestive Endoscopy, or the Korean Society of Coloproctology, based on the time of attending an academic conference or training education and the number of endoscopies performed;
- Performed more than 300 colonoscopies within the last 2 years.

The Center for K-cospi prepared an information session to provide the prospective participating medical institutions with a general introduction to the pilot study, participation requirements, and application process. At the beginning of the Pilot study, a total of 36 medical institutions (26 primary, 6 secondary, and 4 tertiary institutions) located in Goyang-si and Gimpo-si were selected to participate in the pilot study with 70 endoscopists, who met the requirements. The number of pilot sites, medical institutions, and endoscopists participating in the pilot study have been gradually expanded. As of November 2020, a total of 57 medical institutions (42 primary, 10 secondary, and 5 tertiary institutions) located in Goyang-si, Gimpo-si, and Paju-si with 104 endoscopists are participating in the pilot study.

### Outcome measures

The primary outcomes for colonoscopy effectiveness are (1) the rate of detection of CRC and (2) the stage-shift effect of the colonoscopy procedure. The secondary outcomes are (1) major complications due to colonoscopy and (2) participant satisfaction and attitude towards colonoscopy screening.

### Sample size calculation

A calculated target sample size of 26,640 participants will be required to detect significant differences in the rates of cancer detection between FIT and colonoscopy. This is based on an estimated cancer detection rate of 1.2 per 1000 screened with FIT and 2.2 per 1000 screened with colonoscopy.

### Participant recruitment

Posters and leaflets have been placed in each participating medical institution, main bus and subway stops, and at public health centers within the pilot sites to raise awareness of the pilot study. In addition, the pilot study has been introduced to local newspapers of the pilot sites.

Potential participants are identified from the participating medical institutions during their visits and invited to participate if eligible. In addition, those who express interest in participating in the pilot study to the Center for K-cospia re guided to the nearest participating medical institution after eligibility screening. Table 1 lists the inclusion and exclusion criteria.

**Table 1:** Eligibility criteria

Inclusion criteria	Exclusion criteria
Males and females aged 50–74 years	Subjects who have been diagnosed with CRC or are currently undergoing treatment for CRC
Subjects who live within the pilot sites	Subjects underwent colonoscopy screening within the last 5 years

Subjects are allowed to participate in the pilot study only once. Recruitment was initiated in August 2019 and will continue until the planned sample size is reached.

### Informed consent

Eligible participants are provided with comprehensive information material, which includes the aims and procedures of the pilot study, data collection, and privacy policy by the participating medical institutions. If the eligible participants decide to participate in the pilot, they have to provide written informed consent to the Center for K-cospi as study subjects. The participants are informed that they can withdraw from the pilot study at any time for any reason. Data for subjects who withdraw from the study are discarded in a timely manner.

### Screening procedures and follow-up processes

**Fig. 1:**
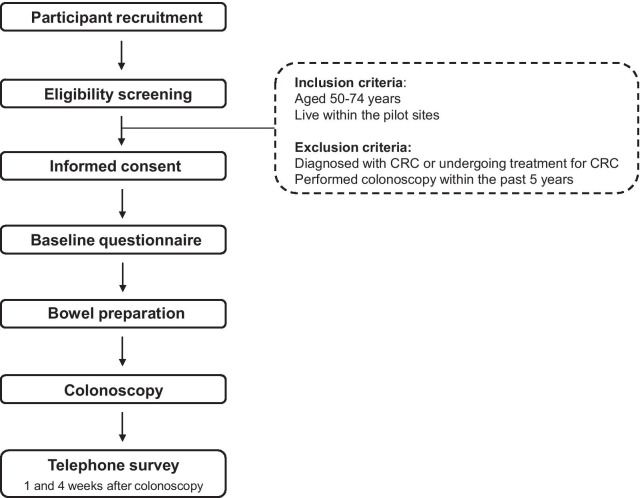
Flow chart of Korean colonoscopy screening pilot study procedure. *CRC* colorectal cancer

The screening procedure is described in Fig. 1. Table 2 shows the SPIRIT schedule of enrolment, interventions, and assessment of this study protocol. At the participating medical institutions, bowel preparation medication is given to participants and a colonoscopy screening date is scheduled. All participants need to complete baseline questionnaires on demographics, current physical symptoms, past medical/surgical history, family history, CRC screening history, medication, and life style before colonoscopy screening.

**Table 2:** Schedule of enrolment, interventions, and assessments

Time point	Study period				
	Enrolment	Pre-treatment	Treatment	1-week follow-up	4-week follow-up
Enrolment					
Eligibility screen	X				
Informed consent	X				
Baseline questionnaire		X			
Interventions					
Bowel preparation		X			
Colonoscopy			X		
Assessments					
Telephone survey				X	X

The participants are asked to visit the medical institution on a scheduled date after their bowel preparation. Polyethylene glycol (PEG) 4L, PEG 2L, and oral sulfate solution are used as standard bowel preparation regimens in the study. All colonoscopies are performed by the participating endoscopists at each participating medical institution with or without sedation, according tothe preference of the participant. The local pathology labs or pathology labs within the participating medical institutions are responsible for tissue examination.

Endoscopists are responsible for follow-up workups or referral of participants for further investigations following their usual referral process, if necessary. Endoscopists need to provide detailed information to the Center for K-cospi on the screening results, results of additional workup, or details of referral if any.

The participants are informed to consult the hospital or visit the nearest emergency room if they experience any uncomfortable symptoms or complications after the colonoscopy procedure. The Center for K-cospi conduct a telephone survey twice—first within 7 days and the next within 4 weeks after colonoscopy—to collect data on any post-colonoscopy complications such as pain, bleeding, and perforations, which could have occurred and the level of satisfaction of the participants with the colonoscopy screening.

### Data collection and management

The results of individual participants for the baseline questionnaire, colonoscopy, and pathology are stored in an electronic case report form on secure servers, which can be accessed only by authorized staff at the participating medical institutions. Participants areas signed a unique de-identified study ID and no personal information are stored in the electronic database. The centralized electronic case report form allows the Center for K-cospito continuously monitor the number of scheduled appointments, the number of completed screenings, colonoscopy results, and any missing, inadequate, or pending test results. All hard copy material collected at the participating medical institutions are routinely forwarded to the Center for K-cospi and kept in a secure cabinet. This includes the informed consent forms, screening questionnaires, pathology results, and spreadsheets containing personal information.

Data will be stored for a period of 15 years. For further analysis, the data will be linked to hospital medical records where diagnostic tests will be conducted, the cancer registry data of the Korean Central Cancer Registry, and death certificate data of the Korean National Statistical Office based on informed consent and ethical approval. This will be done to monitor the diagnostic workup, subsequent treatment, stage of cancer diagnosis, and CRC incidence and mortality during the 15 years of follow-up.

### Safety monitoring

The Center for K-cospi continuously tracks the occurrence of any immediate or delayed serious complications arising from colonoscopy depending on the information reported directly by the participating medical institutions or obtained from participants through telephone surveys. If we identify severe adverse events or unexpected admissions after colonoscopy, the Center for K-cospi requests a detailed investigation of the medical records from the hospital where the complications were treated. Moreover, it reports those events to the Monitoring Committee, Korean Public Institutional Review Board (IRB), and the National Cancer Center IRB. Early termination of the pilot study can be considered if the rate of severe adverse events is higher than expected, i.e.,1 cases per 1000 screening, 4 cases per 1000 screening, and 0.07 cases per 1000 screening for perforation, severe bleeding, and death, respectively. The Center for K-cospi has contracted an insurance to cover study-related harm.

### Quality control and quality assurance

The Center for K-cospi prepared for an educational session at the beginning of the pilot study for the participating endoscopists to provide them with information to improve their quality of the colonoscopy procedure. Participating physicians were recommended to keep an average withdrawal time of at least 6 min to fully observe lesions and not to perform more than 5 colonoscopies for pilot study per day for quality control. In addition, a manual for the pilot study was developed and distributed to the participating medical institutions to ensure the pilot study procedure complies with the study protocol. The Center for K-cospi will thoroughly evaluate each colonoscopy with the following quality indicators: the quality of the bowel preparation, cecal intubation rate, withdrawal time, and adenoma detection rate. Furthermore, the Center for K-cospi will conduct regular audits for documentation and video recording of randomly sampled colonoscopies to ensure that guidance to enhance colonoscopy quality is being followed. The Monitoring Committee will be provided with a report on the quality analyses.

### Data analysis plan

Colonoscopy performance, including CRC detection rate and stage-shift, will be reported during the whole study period. Participant data will be matched against the national CRC screening program database of the National Health Insurance Service to compare the performance of the colonoscopy with that of the FIT. After completion of our study, we will analyze the long-term effects of colonoscopy screening, such as CRC mortality and incidence reduction, and its cost-effectiveness by linking data with the Korean Central Cancer Registry and death certificate data of the Korean National Statistical Office. In addition, complications related to colonoscopy over the study period, and satisfaction of participants will also be described. Missing values will not be imputed. The Center for K-cospi will conduct all analyses on de-identified data.

### Ethical considerations

This pilot study and its protocols have been approved by the Korean Public IRB and the National Cancer Center IRB (IRB number: P01-201908-11-003; P01-202004-11-005; P01-202004-11-006; P01-202011-11-001; NCC2019-0162). The study will be conducted in compliance with the approved protocol and any deviations from the protocol will not be executed without prior review and approval from the IRB.

### Dissemination of results

The findings of this pilot study will be published in peer-reviewed scientific journals and presented at national and international conferences. The results will be published in a statistical aggregate form so that no individual information will be identifiable.

## Discussion

In this paper, we presented the objective, design, and study protocol of K-cospi. The present pilot study is planned to examine the effectiveness, complications, feasibility, and acceptability of colonoscopy as a primary method for CRC screening. FIT is provided free-of-charge as the primary CRC screening method through the NCSP in the Republic of Korea. However, their CRC screening rate was as low as 25% in 2012, and it was the lowest compared to their screening rates for other cancer types, such as stomach, liver, breast, and cervix [7]. In addition, a recent survey found that 68.7% of the participants would prefer the NCSP to adopt colonoscopy as its primary CRC screening test because it is accurate and can provide therapeutic options [6]. K-cospi was launched at an opportune time considering the low CRC screening rates of NCSP and the growing demand for colonoscopy by the national CRC screening program. Furthermore, this pilot study is of importance given that the lack of studies about the appropriateness of colonoscopy as the primary modality of CRC screening.

Previous studies have evaluated the efficacy and disadvantages of colonoscopy screening. A systematic review of observational studies of endoscopy demonstrated that the CRC incidence reduced by 69% (relative risk [RR] 0.31; 95% confidence interval [CI] 0.12–0.77) and mortality reduced by 68% (RR 0.32; 95% CI 0.23–0.43) [8]. Elmunzer et al. [9] found that colonoscopy was more effective than guaiac fecal occult blood test (gFOBT) (RR 0.49; 95% CI 0.30–0.76) in reducing CRC mortality. In addition, the detection rates for advanced adenoma with colonoscopy was significantly higher than those with gFOBT or FIT [10, 11]. However, the mortality rate within 30 days after endoscopy, major bleeding rate, and perforation rate were estimated to be about 1 in 15,000, 0.8 in 1,000, and 0.07–0.4 in 1000 procedures, respectively [12–15]. In other words, colonoscopy is vastly effective in screening for CRC but is highly risky at the same time. Therefore, it is important to evaluate the benefits of colonoscopy screening against its disadvantages in terms of efficacy, process, and cost. Cost is a decisive criterion for the implementation of a screening intervention [16].

Currently, there are four on-going large-scale randomized controlled trials that compare colonoscopy with no screening or fecal immunochemical testing for CRC incidence or mortality in 10–15 years [17]. The trials were launched between 2009 and 2014, and the results of these trials will presumably be presented in 10 years. Therefore, findings from the present pilot study regarding the suitability of colonoscopy as the primary method for CRC screening in organized population-based screening programs are necessary to provide critical insights into the future direction of national CRC screening program in the Republic of Korea.

The protocol of this pilot study has some limitations that should be noted. First, we will not randomizing participants into the two study arms. It is difficult to carry out a randomized controlled trial because FIT is provided free-of-charge every year through the Korean NCSP. To overcome this shortcoming in the study design, the participants will be matched according to their gender and age with the data from the Korean NCSP for CRC to compare the performances of FIT and colonoscopy. Second, the generalizability of the study findings will be limited because the participation rate in this pilot study, the characteristics of participants, and the clinical setting of medical institutions may vary depending on regional characteristics. To minimize this limitation, we selected pilot sites that represent the CRC epidemiology of the whole country and have a balanced characteristic of urban and rural areas. In addition, we evenly included first, second, and tertiary hospitals so that the study can be conducted under real-world circumstances.

In summary, the findings of this pilot study will provide critical information regarding the acceptability and feasibility of colonoscopy as the primary modality of the Korean National Cancer Screening Program. They will lead to more effective and efficient screening programs for CRC.

### Trial status

- Protocol version 2.5 as of November26, 2020
- The date recruitment began: August 8, 2019
- Approximate date when recruitment will be completed: December 31, 2024

## Data Availability

The datasets used/analyzed in this study are available from the corresponding author on reasonable request.

## Abbreviations

CRC: Colorectal cancer
FOBT: Fecal occult blood test
NCSP: National Cancer Screening Program
FIT: Fecal immunochemical test
PEG: Polyethylene glycol
IRB: Institutional review board
gFOBT: Guaiac fecal occult blood test
RR: Relative risk
CI: Confidence interval
